# Biochemical and structural characterization of analogs of MRE11 breast cancer-associated mutant F237C

**DOI:** 10.1038/s41598-021-86552-0

**Published:** 2021-03-29

**Authors:** Samiur Rahman, Mahtab Beikzadeh, Michael P. Latham

**Affiliations:** Department of Chemistry and Biochemistry, Texas Tech University, Lubbock, TX 79409-1061 USA

**Keywords:** NMR spectroscopy, DNA repair enzymes

## Abstract

The MRE11–RAD50–NBS1 (MRN) protein complex plays a vital role in DNA double strand break sensing, signaling, and repair. Mutation in any component of this complex may lead to disease as disrupting DNA double strand break repair has the potential to cause translocations and loss of genomic information. Here, we have investigated an MRE11 mutation, F237C, identified in a breast cancer tumor. We found that the analogous mutant of *Pyrococcus furiosus* Mre11 diminishes both the exonuclease and endonuclease activities of Mre11 in vitro. Solution state NMR experiments show that this mutant causes structural changes in the DNA-bound Mre11 for both exo- and endonuclease substrates and causes the protein to become generally more rigid. Moreover, by comparing the NMR data for this cancer-associated mutant with two previously described Mre11 separation-of-nuclease function mutants, a potential allosteric network was detected within Mre11 that connects the active site to regions responsible for recognizing the DNA ends and for dimerization. Together, our data further highlight the dynamics required for Mre11 nuclease function and illuminate the presence of allostery within the enzyme.

## Introduction

DNA damage is a frequent and natural event that occurs in all cells. The DNA damage response (DDR) is therefore crucial for maintaining the stability and fidelity of genetic information. The DDR requires the action of many proteins to detect, signal, and repair the damage; accordingly, problems in any of the repair machinery can lead to mutations that may cause disorders characterized by genome instability and/or cancer^1–3^. Different forms of DNA damage can occur to the genome (e.g., modifications to the nucleobase or ribose-phosphate backbone), and among these, DNA double strand breaks (DSBs) are the least common but most dangerous form^4^. Correspondingly, the cell has developed a number of strategies to repair DNA DSBs, with homologous recombination (HR) and non-homologous end joining (NHEJ) serving as the two main repair pathways^5,6^.

The essential MRE11-RAD50-NBS1 (MRN) protein complex is one of the first responders to a DNA DSB and is required in both HR and NHEJ^7,8^. MRE11 is a homodimer and functions as a Mn^2+^-dependent 3′-to-5′ exonuclease and ssDNA endonuclease^9,10^. These nuclease functions are associated with generating the 3′-overhangs necessary for HR^11^ and also extend to the removal of DNA–protein adducts^12–14^. Each MRE11 binds to a RAD50, which is a member of the ATP binding cassette (ABC) superfamily of ATP hydrolyzing enzymes^15,16^. Together, the MRE11_2_-RAD50_2_ (or simply MR) assembly forms the evolutionarily conserved core complex. The ATP binding and hydrolyzing activities of RAD50 control the overall activity of the complex. In the stable ATP-bound conformation, the two RAD50 protomers associate, occlude the MRE11 active sites, and form a central DNA binding groove that is important for telomere length maintenance^17,18^. In the ATP-free conformation, the two RAD50s are dissociated, and MRE11 can bind and hydrolyze the DNA phosphodiester backbone^19^. The cycling of MR conformations is required for DNA unwinding^17,20^, processive MRE11 nuclease activity^21^, and downstream signalling through ATM kinase^22,23^. Lastly, NBS1 (or Xrs2 in *S. cerevisiae*), which also binds to MRE11, is a eukaryotic specific scaffolding protein that binds to select phosphorylated proteins, targeting them to the site of the DNA DSB^24,25^.

**Figure 1:**
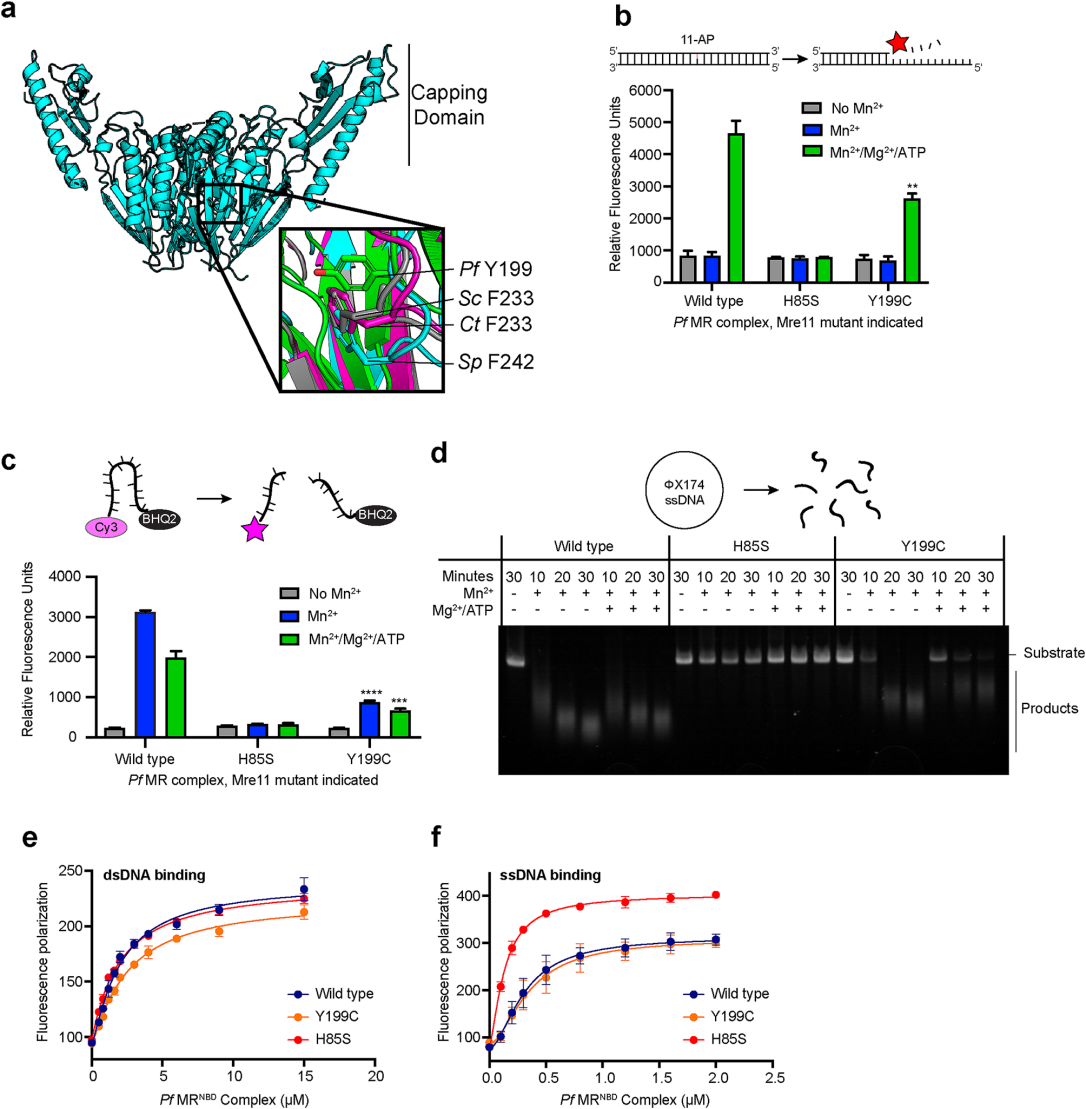
*Pf* Mre11 Y199C has deficient nuclease activity. (**a**) X-ray crystal structure of *S. pombe* Mre11^ND^. Outset shows the superimposition of a region of the X-ray crystal structures for *P. furiosus*^32^, *S. pombe*^66^, and *C. thermophilum*^67^ Mre11^ND^ shown in green (PDB ID: 1II7), cyan (4FCX), and magenta (4YKE), respectively. The S. *cerevisiae* structure, shown in grey, is from a homology model that was relaxed via molecular dynamics simulations^68^. Structurally similar residues to *H. sapiens* F237C are shown in sticks. (**b**) Grey, blue, and green bars indicate the 2-AP fluorescence resulting from MR exonuclease activity (30 min at 60 °C) in the no Mn^2+^, Mn^2+^, and Mn^2+^/Mg^2+^/ATP conditions, respectively. The two asterisks (**) denote a p-value of less than 0.01 compared to wild type activity. Schematic above illustrates the exonuclease reaction. (**c**) Quantification of endonuclease activity (30 min at 60 °C) using the Cy3/BHQ2-labeled ssDNA substrate. Colors are same as in (**b**). Three (***) and four (****) asterisks denote a p value of less than 0.001 and 0.0001, respectively. Schematic above illustrates the endonuclease reaction. (**d**) Agarose gel showing the endonucleolytic degradation of a ΦX174 single stranded virion circular DNA after incubation with MR complexes at 60 °C in Mn^2+^ or Mn^2+^/Mg^2+^/ATP for the indicated amount of time. Mn^2+^ was omitted as a negative control. Schematic above illustrates the plasmid-based endonuclease reaction. (**e** and **f**) Plots of the fluorescence polarization of a labeled (**e**) dsDNA or (**f**) ssDNA substrate versus wild type (blue), Y199C (orange), and H85S (red) *Pf* MR^NBD^ concentration. The solid line is the fit to the data as described in the Material and Methods, whereas the points and error bars are the average and standard deviation of three measurements.

Mutations in MRE11 have been associated with several malignancies^26^. For example, hypomorphic MRE11 mutations give rise to the autosomal recessive ataxia telangiectasia-like disorder (ATLD), which is characterized by sensitivity to ionizing radiation, immunodeficiency, and neuronal and cerebellar degeneration^27^. Mutations to MRE11 are also linked to a variety of cancer types broadly characterized by chromosomal instabilities^28–30^. The Phe237Cys (F237C) mutation in human MRE11 is one such mutation that was found in a breast cancer tumor sample^28^. In the crystal structure of the human MRE11 nuclease and capping domains, the aromatic ring of Phe237 is located in the conserved hydrophobic core of the nuclease domain and is surrounded by the side-chains of Asn212, Ile238, and Asp235 and takes part in a ring stacking interaction with Tyr179 (ref. 31). Thus, it was initially hypothesized that this mutation would disrupt the structure of MRE11 (ref. 31). Here, we describe our efforts to further characterize the effects of this mutant on the structure and function of MRE11. For our in vitro studies, we used the well-studied MR complex from the hyperthermophilic archaea *Pyrococcus furiosus (Pf)* as a model. Structure-based alignment suggests that an aromatic residue is conserved at this position in eukaryotes (Fig. 1a; *S. pombe* Phe242 (F242) and *C. thermophilum* Phe233 (F233); PDB IDs 4FCX and 4YKE, respectively). In *Pf*, there is a tyrosine at the corresponding position (Tyr199), and similar packing interactions are observed around this aromatic ring (PDB ID 1II7)^32^. The high level of structural conservation across the domains of life implies that the aromatic residue at this position in Mre11 is important for function. Therefore, *Pf* Mre11 Tyr199 was mutated to cysteine to produce the corresponding cancer-associated mutant for use in the biochemical and structural studies reported below. We demonstrate that *Pf* Mre11 Tyr199Cys (Y199C) has decreased exo- and endonuclease activities in vitro, and solution state nuclear magnetic resonance (NMR) spectroscopy data suggest that this decreased catalytic activity originates from altered structure and dynamics within Mre11 that are critical for proper orientation of DNA substrates. Using this and our previously published NMR data on other nuclease deficient mutants, we were able to propose an allosteric network within Mre11. We also made the analogous mutation in *S. cerevisiae* (F233C) to examine the effect of the mutation in budding yeast and show that this mutation has no effect on DNA DSB repair in *S. cerevisiae* consistent with other nuclease mutants.

## Results

### *Pf* Mre11 Y199C affects both exo- and endonuclease activities

The Mn^2+^-dependent Mre11 exonuclease activity of *Pf* Mre11–Rad50 complexes (M_2_R_2_ or simply MR) was measured using a 2-aminopurine (2-AP) based fluorescence assay. We compared the exonuclease activity of the Y199C MR complex with wild type and His85Ser (H85S) MR complexes (Fig. 1b). Mre11 H85S was used as negative control as it was previously shown to be both exo- and endonuclease inactive^33,34^. As expected, no activity was observed for any of the constructs in the absence of Mn^2+^. Since ATP hydrolysis by Rad50 is required for processive exonuclease activity^21^, we assayed the exonuclease activity in both the presence and absence of ATP. Without ATP, none of the MR complexes showed Mn^2+^-dependent exonuclease activity; however, with ATP/Mg^2+^, we observed differences in the resulting relative 2-AP fluorescence of wild type, H85S, and Y199C MR. The Y199C mutant showed a significant (p < 0.01) reduction of exonuclease activity in comparison to wild type, whereas H85S was completely inactive as expected (Fig. 1b).

Next, we sought to determine the effect of the Y199C mutant on Mre11 ssDNA endonuclease activity. Using a Cy3/BHQ2 fluorescence-based quantitative endonuclease assay, we observed Mn^2+^-dependent activity for wild type MR that is not dependent on ATP/Mg^2+^ (Fig. 1c). When comparing the endonuclease activity across the mutants, we observed a significant (p < 0.01), albeit not complete, decrease in function for Y199C MR and no cleavage for H85S MR. To confirm these results, we also monitored the endonuclease activity of the MR complexes against a ssDNA virion plasmid substrate over time (Fig. 1d). In reactions containing Mn^2+^, both wild type and Y199C produced smaller DNA fragments that migrated faster through an agarose gel, indicating successful digestion of the plasmid, whereas H85S gave only intact DNA bands. ssDNA fragments from Y199C cleavage did not migrate as far through the gel as compared to wild type generated fragments revealing that Y199C produces larger endonuclease products than wild type. Thus, the Mre11 Y199C mutant has defects in both exo- and endonuclease activities.

To determine if the effects we observed in exo- and endonuclease activities in the Y199C mutant were due to the inability to bind to substrate DNAs, we determined the binding affinities, via the change in fluorescence anisotropy of labeled DNA, for MR^NBD^ complexes (where the Rad50 nucleotide binding domain construct lacks the majority of the coiled-coil and Zn^2+^ hook domains) in the absence of ATP/Mg^2+^. Under these conditions, the observed DNA binding is from Mre11^15^. For both exo- (dsDNA, Fig. 1e) and endonuclease (ssDNA, Fig. 1f) substrates, we observed similar K_D_s for wild type and Y199C MR^NBD^ complexes: 2.0 and 2.5 μM, respectively for dsDNA and 0.31 and 0.35 μM, respectively for ssDNA. Thus, differences in nuclease function do not arise from the general inability to bind to substrate DNA.

### Y199C mutant affects *Pf* Mre11 protein structure and dynamics

**Figure 2:**
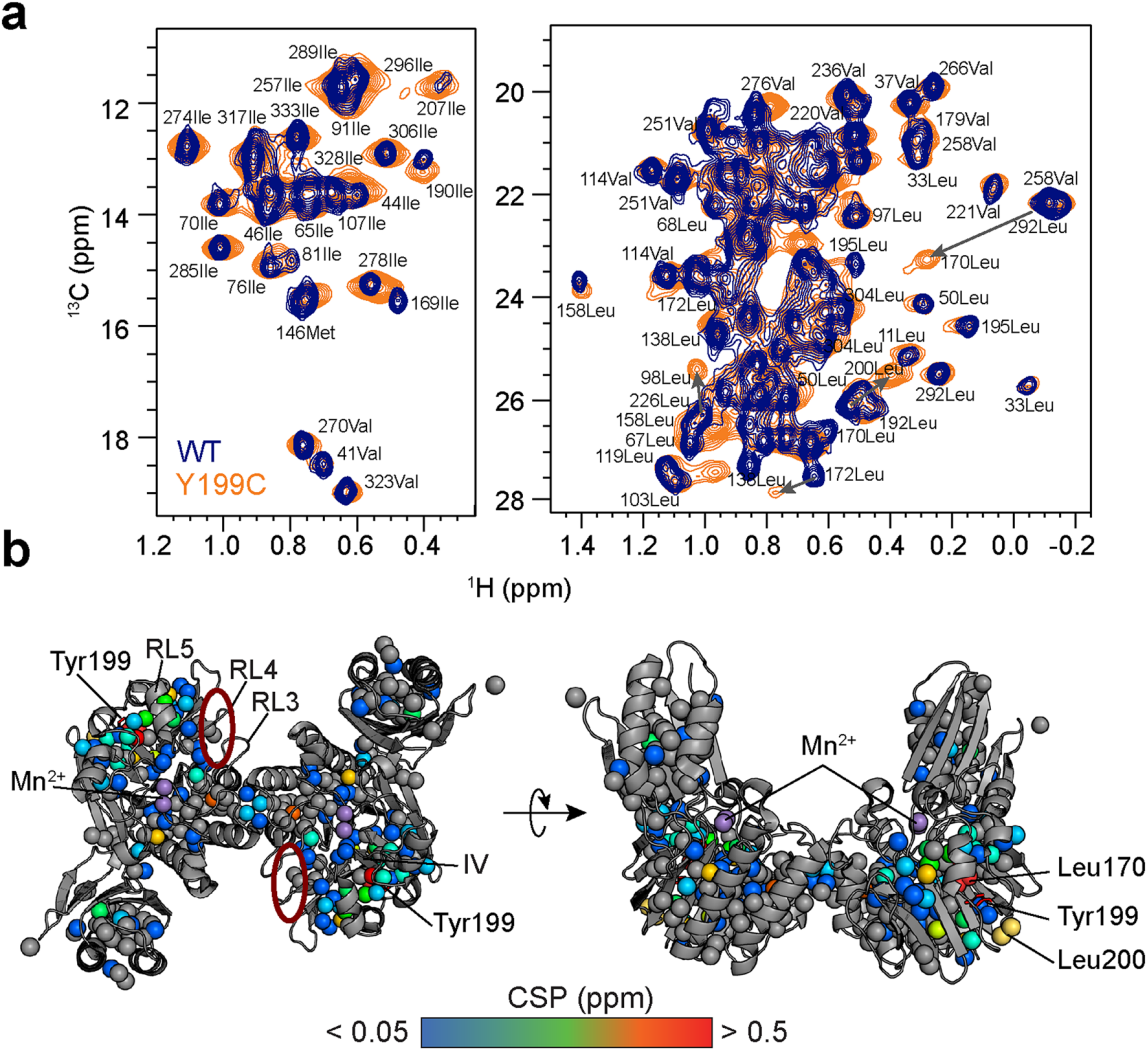
*Pf* Mre11 Y199C causes NMR chemical shift perturbations throughout the protein. (**a**) Overlays of wild type (blue) and Y199C (orange) *Pf* Mre11^ND^ 2D ^13^C, ^1^H methyl-TROSY NMR spectra. The two overlays are of different regions in the spectra. Peak assignments of many methyl groups are indicated, and grey arrows indicate the position of highly shifted peaks upon mutation. (**b**) The magnitude of side-chain methyl group CSPs between wild type and Y199C are colored on the structure of *Pf* Mre11^ND^ (PDB ID: 1II7) according to the associated gradient. Grey spheres are methyl groups that did not experience significant CSPs. The position of various residues, structural motifs, and the catalytic Mn^2+^ ions (purple spheres) referred to in the text are indicated. A red circle indicates the position of the conserved basic patch.

To understand the structural effects of the *Pf* Mre11 Y199C mutant, we turned to solution state NMR spectroscopy. Because of the large size of the Mre11 construct used here (Mre11 nuclease and capping domains dimer, Mre11^ND^, ~ 65 kDa), we utilized uniformly deuterated, side-chain methyl group^13^ CH_3_-labeled samples and methyl-Transverse Relaxation Optimized SpectroscopY (TROSY)^35,36^. First, we compared the apo form (i.e., Mn^2+^-free and DNA-free) of Mre11^ND^ Y199C with wild type. The side-chain methyl group resonances in the two-dimensional (2D) methyl-TROSY ^13^C, ^1^H correlation spectra revealed that the vast majority of the peaks overlay for the two constructs (Fig. 2a), indicating that the global fold of Mre11^ND^ is unchanged in the mutant. The largest chemical shift perturbations (CSPs) were observed for Leu170 and Leu200 (Fig. 2b), which pack above and below Tyr199. These large peak movements were expected due to the altered chemical environment resulting from the loss of the aromatic residue in Y199C. Other large CSPs were observed for residues in the DNA recognition loop 5 (RL 5), catalytic motif IV, and near the Mre11 dimerization interface (Fig. 2b). There were smaller CSPs observed around RL 3 and 4, which are in a conserved basic patch involved in DNA binding^37^. Other small CSPs were observed in the capping domain which is ~ 46 Å away from the site of the mutation.

**Figure 3:**
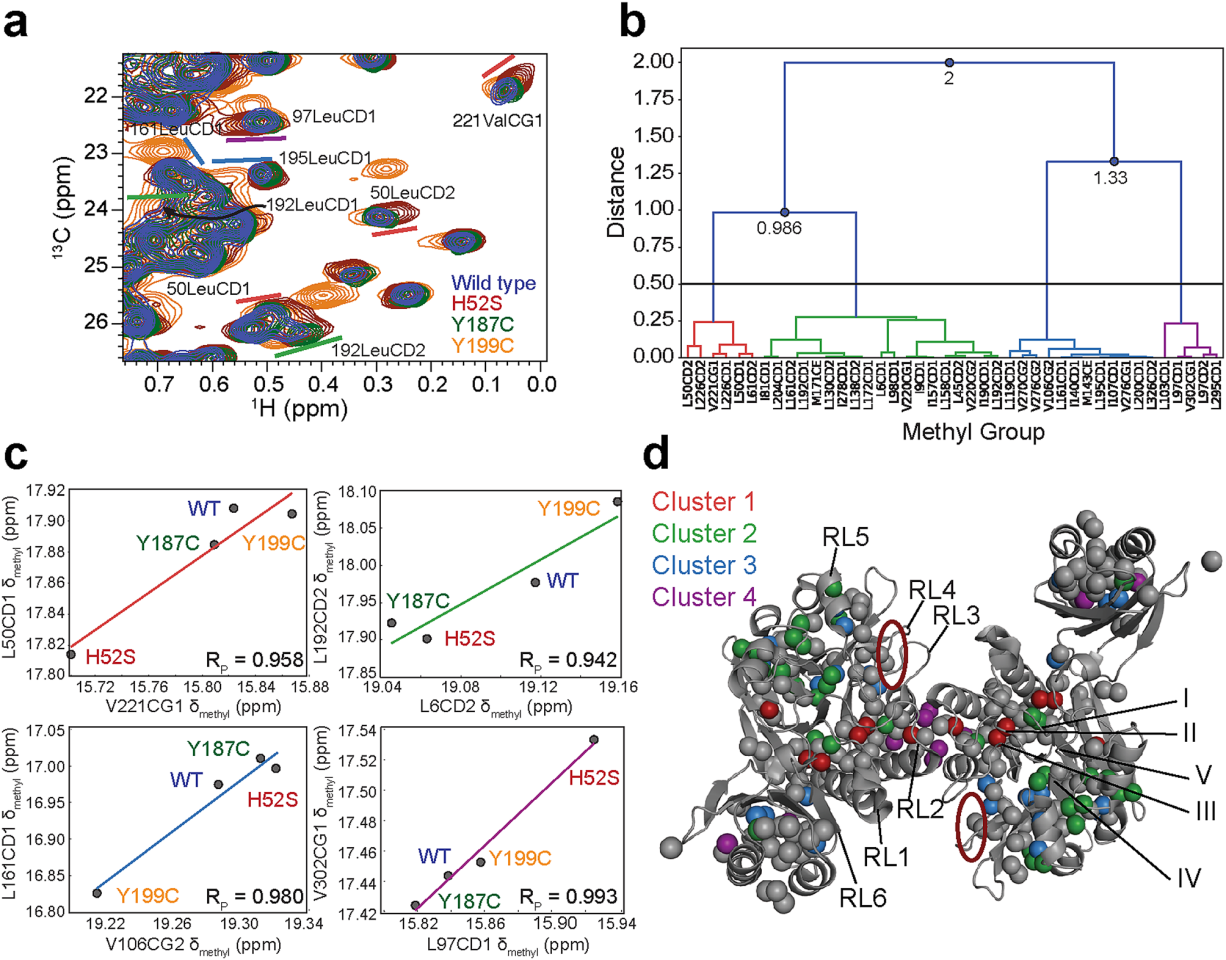
CHESCA reveals a potential allosteric pathway in Mre11^ND^. (**a**) Overlay of 2D ^13^C, ^1^H methyl TROSY NMR spectra for wild type (blue), H52S (red), Y187C (green), and Y199C (orange) *Pf* Mre11^ND^. Peak assignments are given for several peaks that were considered for CHESCA. The colored bars correspond to the cluster where that peak is located (see (**b**) and (**d**)). (**b**) Dendrogram of methyl groups determined by CHESCA. A distance cut-off of 0.5 (horizontal black line) was used to define the clusters. Clusters 1, 2, 3, and 4 are colored red, green, blue, and purple, respectively. (**c**) Representative pairwise inter-residue correlation plots of the combined chemical shifts (δ_methyl_) for each of the four clusters. The Pearson’s correlation coefficient (R_P_) is given in the lower right corner. (**d**) Structure of *Pf* Mre11^ND^ highlighting the side-chain methyl groups whose CSPs from mutation cluster according to CHESCA. Methyl groups not clustered are colored grey. DNA recognition loops (RL) and conserved catalytic motifs (I–V) are indicated. The location of the conserved basic patch is outlined by the red circle.

We have previously biochemically and structurally characterized two *Pf* Mre11 separation-of-nuclease function mutants: H52S and Y187C^34^. The NMR data collected on those mutants also showed CSPs for side-chain methyl groups around the catalytic motifs and dimerization interface. Since H52S, Y187C, and Y199C have similar biochemical (i.e., loss of exonuclease activity) and structural phenotypes, we questioned whether or not any changes in the structure were common among these mutants. An overlay of the methyl-TROSY spectra for wild type and the three mutants (Fig. 3a) showed that several resonances throughout *Pf* Mre11^ND^ move in a linear fashion due to mutations, yet the pattern of the peak movements were not the same for all methyl groups (e.g., 97LeuCD1 and 195LeuCD1 in Fig. 3a). Nevertheless, linear movement as observed here can be indicative of a shift in the population between two states. We therefore performed chemical shift covariance analysis (CHESCA), a method for clustering residues undergoing the same populated weighted average change in peak position because of a perturbation (here, the perturbation is mutation)^38,39^. For CHESCA, the pattern of the four peak positions for a given methyl group (i.e., the peak for the same methyl group in wild type, H52S, Y187C, and Y199C) is compared to the pattern observed for every other methyl group; methyl groups with high correlation (low distance in Fig. 3b) in peak movement patterns are then clustered together. These clusters often uncover allosteric networks within proteins^38,40–42^. CHESCA of *Pf* Mre11^ND^ resonances with significant changes in peak position upon mutation resulted in four clusters of residues (Fig. 3b and 3d). As expected, methyl groups within a cluster have peak movement patterns that are highly correlated with the peak movement patterns from other members of that cluster (Fig. 3c) but not with those from methyl groups in other clusters. These four clusters map to a line of residues between active site motifs II and III and the dimerization interface (cluster 1), an area of Mre11 between Y187 and Y199, which encompasses regions of the protein that may recognize dsDNA ends (cluster 2), various residues throughout the structure (cluster 3), and the dimerization interface (cluster 4) (Fig. 3d).

**Figure 4:**
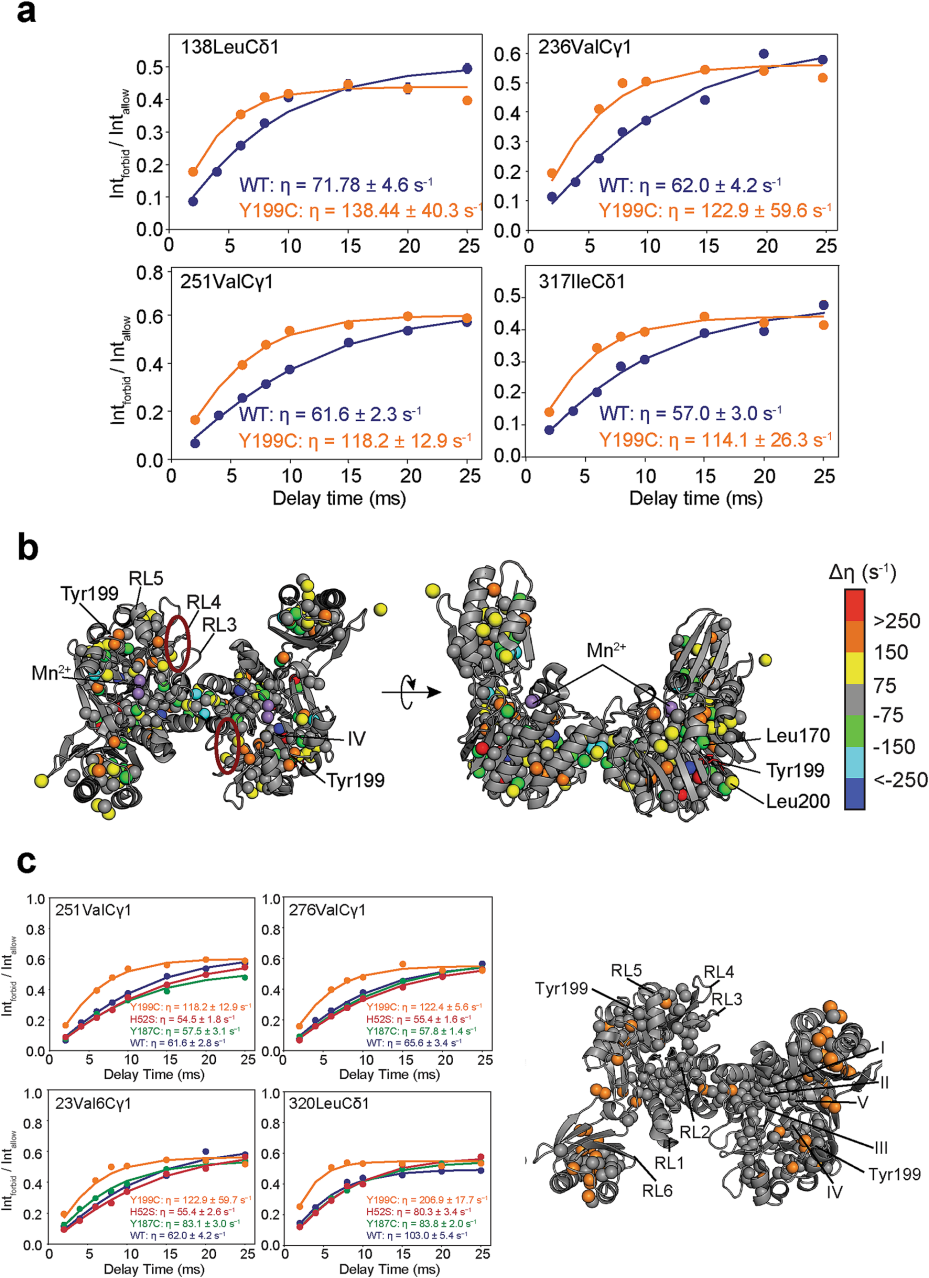
*Pf* Mre11 Y199C decreases the flexibility of the nuclease and capping domains. (**a**) Representative ^1^H–^1^H cross-correlated “forbidden” triple-quantum relaxation curves. The data for wild type and Y199C are colored blue and orange, respectively. Error in the data points is derived from the noise in the spectra. The solid lines represent the fit of the data as described in the Material and Methods. Calculated η rates are given in the lower right corners. (**b**) Δη between wild type and Y199C are mapped on the structure of *Pf* Mre11^ND^ and are colored according to the associated gradient. The position of various residues, structural motifs, and catalytic Mn^2+^ ions referred to in the text are indicated. A red circle indicates the position of the conserved basic patch. (**c**) Representative ^1^H–^1^H cross-correlated “forbidden” triple-quantum relaxation curves for wild type (blue), H52S (red), Y187C (green), and Y199C (orange). Side-chain methyl groups that have larger η rates in only the Y199C mutant are highlighted in the structure of *Pf* Mre11^ND^ as orange spheres.

We next used the side-chain methyl group ^13^CH_3_-labeled *Pf* Mre11^ND^ NMR samples to compare the wild type and mutant side-chain methyl group dynamics on the ps-ns timescale at 50 °C. These motions were characterized by measuring the cross-correlated relaxation rates (η) of methyl ^1^H–^1^H “forbidden” triple quantum coherences. η describes the ps-ns timescale amplitude of the side-chain methyl group motion and global tumbling^43^. Because there is no evidence that Y199C alters dimerization of Mre11, we interpreted changes in η rates as differences in side-chain motion and not changes in global tumbling. Figure 4a shows representative relaxation curves for individual side-chain methyl groups highlighting the differences observed in the η rates upon Y199C mutation. Figure 4b shows the differences in the η relaxation rates (Δη = η_MUT_ − η_WT_) mapped onto the crystal structure of *Pf* Mre11^ND^. The data show that Y199C generally leads to Δη > 0 (average Δη rate was 27.2 s^−1^), which indicates decreased side-chain methyl group flexibility throughout the entire structure. Specifically, we observed the largest Δη values for methyl groups adjacent to the site of the mutation as well as in the capping domain and the basic patch, which mirror the changes observed above in the CSPs. Collectively, the NMR data show that the immediate area around the site of the mutation was predominantly affected by Y199C, as expected, and that perturbations to the conserved basic patch, a region suggested to be important for proper positioning of the exonuclease substrate, could be in part what is causing the lower exonuclease activity of the mutant.

We also compared η rates across wild type, Y199C, and the two separation-of-nuclease function *Pf* Mre11^ND^ mutants (Fig. 4c). Here, we noted that several side-chain methyl groups in the Y199C mutant had a significant deviation from the η rates observed for wild type, H52S, and Y187C. When the side-chain methyl groups with deviant η rates were mapped onto the structure of *Pf* Mre11^ND^ (Fig. 4c, *right*), we observed a clustering of residues in the capping domain and linkers connecting the nuclease and capping domains—regions consistent with the proposed ssDNA binding site^33^. Therefore, differences between the endonuclease activities of the Y199C, which has impaired activity (Fig. 1), and H52S and Y187C, which have wild type-like activity, could be due to differences in the dynamics in this region. Moreover, comparing the pattern of methyl groups with deviant η rates to methyl groups that clustered in CHESCA (Fig. 3d), we noticed very few methyl groups are observed in both sets. This observation supports the notion, exemplified by separation-of-function mutations^33,34^ and inhibitors^44^, that different regions within Mre11 are responsible for exo- and endonuclease functions. Furthermore, the observed structural and dynamic perturbations to side-chain methyl groups distal to the site of mutation (in the nuclease motif and dimerization interface) combined with the CHESCA clusters further substantiate a role for allostery within Mre11.

### Y199C mutant affects *Pf* MRE11 DNA binding

**Figure 5:**
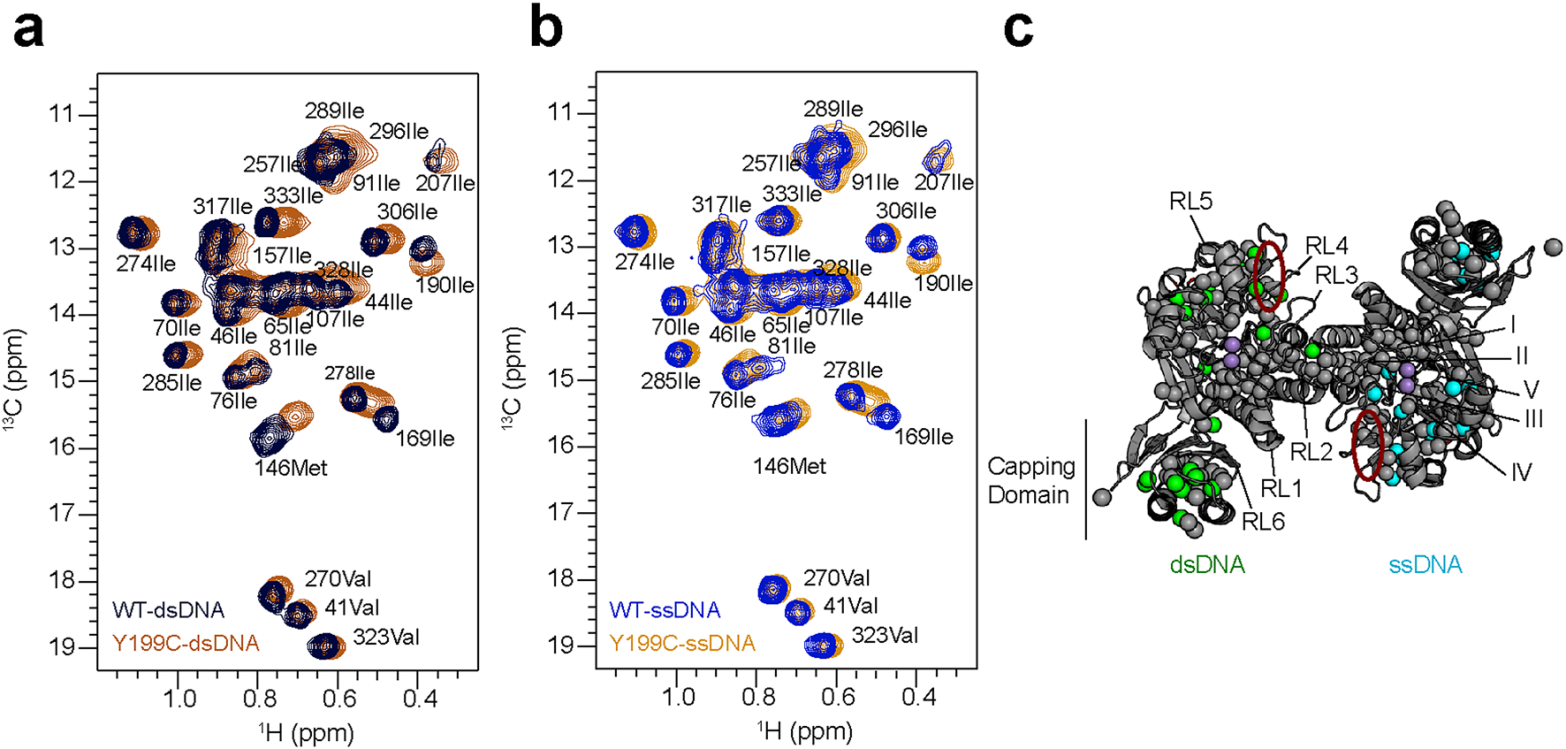
Pf Mre11 Y199C mutant alters the binding of dsDNA and ssDNA. Overlay of a region of the 2D ^13^C, ^1^H methyl-TROSY spectra for (**a**) wild type (dark blue) and Y199C (dark orange) Pf Mre11^ND^ bound to dsDNA and (**b**) wild type (blue) and Y199C (orange) Pf Mre11^ND^ bound to ssDNA. (**c**) ΔCSPs in dsDNA-bound (left monomer/green spheres) and ssDNA-bound (right monomer/light blue spheres) Pf Mre11^ND^ Y199C are shown on the structure of Pf Mre11^ND^. Conserved catalytic motifs (I–V) and DNA recognition loops (RL1–5) are indicated. The red circle indicates the position of the conserved basic patch.

We next added dsDNA and ssDNA to side-chain methyl group ^13^CH_3_-labeled NMR samples of Y199C Mre11^ND^ to determine if the mutant has an effect on the structure of DNA-bound Mre11 (Fig. 5a and b). To identify differences in the DNA-bound structures, we calculated the deviation of the CSPs between the DNA-bound forms of wild type and Y199C Mre11^ND^ (ΔCSP = |CSP_WT-DNA_ − CSP_Y199C-DNA_|). Significant ΔCSPs (i.e., those greater than one standard deviation of the median ΔCSPs) were observed for both dsDNA- and ssDNA-bound forms of Mre11^ND^ and are mapped onto the crystal structure of *Pf* Mre11^ND^ in Fig. 5c (green and cyan spheres, respectively). In general, the number of ΔCSPs was similar for both dsDNA- and ssDNA-bound Mre11. We have previously shown that perturbations in methyl dynamics around a conserved basic patch were not correlated with a decrease in affinity for dsDNA, as measured by fluorescence polarization, but were instead associated with altered stability of the dsDNA substrate and in turn, a decrease in exonuclease activity^34^. A similar situation may be occurring in *Pf* Mre11 Y199C.

Since the Y199C mutant disrupts endonuclease activity, we compared the significant ΔCSPs observed here upon ssDNA binding with those previously observed for H52S and Y187C^34^. We reasoned that side-chain methyl groups that uniquely experienced a difference in ssDNA binding for the Y199C mutant could be related to the disruption of endonuclease activity. Interestingly, ~ 1/2 of the ssDNA-bound ΔCSPs observed for Y199C were unique (i.e., they were not observed in ssDNA-bound H52S or Y187C), and these side-chain methyl groups clustered within the structure to two main regions: above the site of the mutation where the largest CSPs are observed (e.g., Leu170 and Leu172; Fig. 2) and along the base of the central β-sheet. These residues may therefore offer the first clue into which regions of Mre11^ND^ are important for endonuclease activity.

### DNA DSB repair in yeast is unaffected by the breast cancer mutant

**Figure 6:**
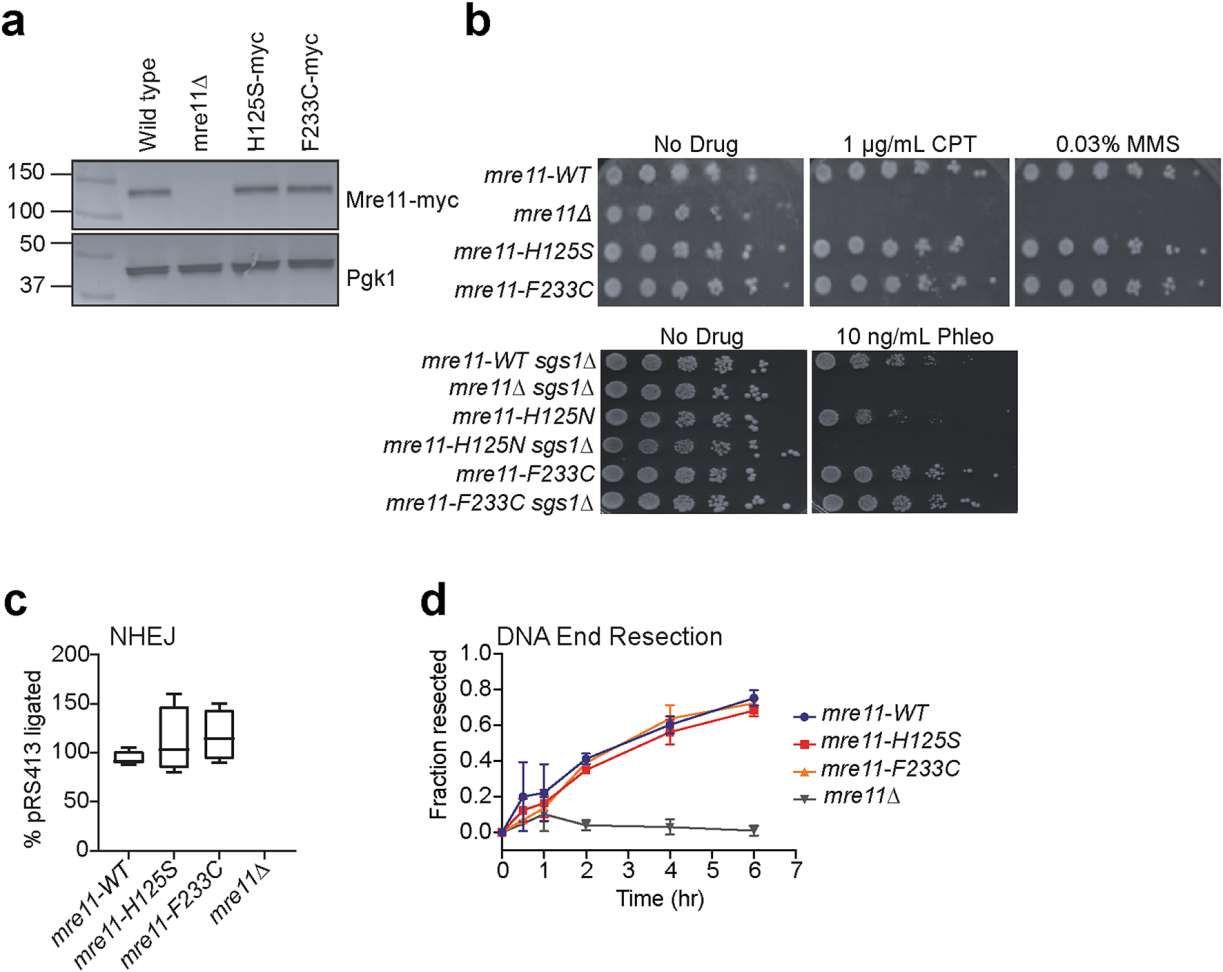
Yeast DNA DSB repair is not affected by the F233C mutant. (**a**) Western blot showing the expression of Mre11-myc protein in wild type, knockout (*mre11Δ*), and mutated *S. cerevisiae*. Phosphoglycerate kinase 1 (Pgk1) was used as a loading control. The gel was cut for western blot against Mre11 and Pgk1, denoted by white space between the panels. The full sections of the gel are shown in Supplementary Figure S1. (**b**) Top, survival assays for serial dilutions of indicated yeast strains on XY plates containing no drug, 1 μg/ml CPT, or 0.03% w/v MMS. Bottom, survival assays for serial dilutions of indicated yeast strains on XY plates containing no drug and 10 ng/mL phleomycin. (**c**) Relative efficiency of DNA DSB repair in the plasmid-based NHEJ assay. Box and whisker plots show the median, high, and low values for each yeast strain. No colonies were observed for the *mre11Δ* strain. Data were normalized to the number of colonies for the wild type *mre11* strain. (**d**) Fraction of DNA with resected ends at a galactose-induced HO endonuclease cut site versus time as measured by real-time PCR. Resection trajectories of each yeast strain are colored as indicated.

Finally, we tested the ability of the analogous mutant in *S. cerevisiae* to perform DNA DSB repair. Myc-tagged *mre11*, *mre11-H125S* (corresponding to nuclease deficient *Pf* Mre11 H85S), and *mre11-F233C* (analogous to *H. sapiens* MRE11 F237C) were stably integrated into the endogenous *mre11* locus of *S. cerevisiae*. Western blots confirmed that mutant Mre11 expression was equal to wild type (Fig. 6a). First, we examined whether or not the analogous cancer-associated mutant was sensitive to the genotoxic agents CPT and MMS, a topoisomerase I inhibitor and a nucleobase alkylating agent, respectively. Figure 6b, top, shows that *mre11*-F233C repairs dsDNA breaks generated by CPT or MMS, as this yeast strain grew as efficiently as wild type and *mre11-H125S* on both the non-treated and drug-treated plates. We also performed survivability assays with phleomycin, which generates ‘clean’ DNA DSBs (i.e., without protein adducts), and as shown in Fig. 6b, bottom *mre11-F233C* repairs this form of DNA damage. To determine if other nucleases (e.g., Dna2 or Exo1)^45–47^ were compensating for the nuclease deficiency of *mre11-F233C* in the DDR, we monitored the sensitivity to phleomycin in a *sgs1*Δ background (Fig. 6b, bottom). As previously shown, the nuclease inactive *mre11-H125N* demonstrated extreme sensitivity to phleomycin when Sgs1, a helicase associated with Dna2, is knocked out^47^, removing a ‘back up’ nuclease from the DDR. On the other hand, *mre11-F233C* again demonstrated wild type ability to survive on phleomycin even in the *sgs1*Δ background (Fig. 6b, bottom). Thus, the remaining nuclease activities of this mutant may be sufficient to maintain the DDR in the absence of Sgs1/Dna2.

Though *mre11-F233C* was not sensitive to genotoxic agents, we were still interested in determining its effectiveness in each of the two major DNA DSB repair pathways. The ability to perform NHEJ was assessed by transforming yeast cells with a linearized plasmid containing a yeast origin of replication and complementary nutritional marker (*HIS3*)^48,49^. Restriction enzyme digestion was performed at a site lacking homology to the yeast genome; therefore, NHEJ is required to repair the DNA DSB and for the yeast to grow on histidine dropout media. *mre11-F233C* showed wild-type levels of NHEJ repair efficiency (Fig. 6c), as did *mre11-H125S*. Lastly, to probe for a defect in HR repair, the DNA end resection efficiency of *mre11-F233C* was assessed via a real time PCR-based assay^50,51^. Kinetic analysis showed *mre11-F233C* exhibited similar levels of end resection as both wild type and *mre11-H125S* at an HO endonuclease induced DNA DSB near the *MAT* locus (Fig. 6d). Thus, *mre11-F233C* mutated yeast are fully capable of performing NHEJ and HR, and these data are consistent with previous reports of Mre11 nuclease activity being dispensable for NHEJ and HR in yeast^52^.

## Discussion

The MRE11 F237C mutation was found in an analysis of ~ 13,000 genes in 11 breast cancers. Additionally, analysis of MRE11 mutations cataloged in cBioportal^53,54^ revealed another mutation at this position (F237L) found in breast invasive ductal carcinoma^55^. In both cases, the mutation at F237 would have been one of many other mutations in various other genes detected in these tumors^28,55^. Therefore, we obviously do not know if these mutations were necessary and/or sufficient to cause the disease. Our in vitro data does show that the analogous mutation in a model Mre11 (*Pf* Mre11 Y199C) has significantly decreased exo- and endonuclease activities and changes the chemical environment of the protein side-chains in both ss- and dsDNA binding regions. Yet, we and others have shown that this phenotype is not sufficient to alter the DNA DSB response in budding yeast^34,52^. Thus, if this spontaneous mutation was an underlying cause for carcinogenesis, it is likely due to the disruption of some other aspect of MRN function, protein–protein interactions, and/or regulation, which occur in humans but not lower organisms.

The data presented here are comparable to our recent study of two other *Pf* Mre11 separation-of-nuclease function mutations: Y187C and H52S^33,34^. Y187C and H52S were exonuclease inactive but endonuclease active, whereas Y199C showed significant reduction in both nuclease activities. NMR data suggests that, like *Pf* Mre11 Y187C and H52S, the Y199C mutant not only changed the structure of Mre11 in the immediate vicinity of the mutated residue but also in functional regions far from the site of the mutation and led to a dramatic overall decrease in side-chain methyl group motions within Mre11. In the case of Y187C and H52S, we determined that the separation-of-nuclease function phenotype is derived from an inability of Mre11 to stabilize the dsDNA exonuclease substrate, and since Mre11 binds to ssDNA and dsDNA differently, exonuclease activity was affected, whereas endonuclease activity was not^34^. Because of the similarity in the NMR data, we propose that a similar phenomenon is occurring in Y199C, though perhaps to a lesser extent as some exonuclease activity is retained in this mutant.

Combining our data here with our previous NMR results for Y187C and H52S allowed us to perform CHESCA (Fig. 3) and uncovered a network of residues in Mre11^ND^ that couples the regions important for DNA binding, and possibly DNA end recognition, with the active site and dimerization interface. X-ray crystal structures and methyl group CSPs from our previous NMR experiments on various Mre11^ND^-DNA complexes have both shown that DNA binding alters the environment of the dimerization interface^34,37^. CHESCA identified a specific set of residues through the core of the Mre11 nuclease domain that might relay DNA binding information to the dimerization motif. We have also examined the effect of these mutants on dsDNA binding (Fig. 5). Interestingly, all of the residues in cluster 1 (red spheres in Fig. 3d) displayed significant deviations from wild type in dsDNA binding (i.e., significant ΔCSPs) in at least two of the three exonuclease deficient mutants we have characterized. This result strengthens the CHESCA output and implies that this network of residues is indeed important for exonuclease activity. Thus, we predict that the mutation of residues within this cluster may also lead to an exonuclease inactive phenotype.

Finally, Y199C disrupts endonuclease activity, whereas the Y187C and H52S mutants were wild type-like. NMR data for Y199C showed some changes within the structure as a result of ssDNA binding which not seen in the other mutants. The residues involved in these changes are unique from those observed upon dsDNA binding and in the CHESCA clusters implying that different networks might exist within Mre11 for relaying ss- versus dsDNA binding information. In support of this, we note that Y199C displayed significant rigidification in the capping domain that was not observed in Y187C or H52S. In conclusion, we have shown through the characterization of nuclease deficient mutants that discrete changes in Mre11 structure and dynamics are critical for exo- and endonuclease activity and likely underlie the separation of the nuclease functions.

## Materials and methods

### Protein expression and purification

Mre11 point mutations were introduced into the expression plasmid using a modified Quikchange approach (Stratagene). Full-length *Pf* Mre11 and truncated *Pf* Mre11^ND^ (amino acids 1–342 comprising the nuclease and capping domains) were expressed by transforming the appropriate plasmid into *E. coli* BL21(DE3) C41 cells (Sigma). For unlabeled protein, cells were grown in LB media, and expression was induced with 1 mM IPTG at 37 °C for 4 h. To prepare U-^2^H, Ileδ1-[^13^CH_3_], Leuδ/Valγ-[^13^CH_3_,^12^CD_3_], Metε-[^13^CH_3_]-labeled *Pf* Mre11^ND^ samples for NMR spectroscopy, cells were grown in deuterated 2 × M9 minimal media^56^ with 1 g/L ^14^NH_4_Cl and 3 g/L ^2^H,^12^C-glucose as the sole nitrogen and carbon sources. 100 mg of each ^13^CH_3_-labeled Ile, Leu, Val, and Met metabolic precursors (Cambridge Isotope Laboratories, Inc)^57^ were added to the media 45 min prior to induction with 1 mM IPTG. Isotopically labeled Mre11^ND^ was then overexpressed for ~ 16 h at 37 °C. In both cases, purification proceeded as previously described^34^.

Full-length and nucleotide binding domain (NBD) constructs of *Pf* Rad50 were expressed and purified as described in Boswell et al.^58^. Mre11-Rad50 complexes were made by mixing equimolar ratios of full-length Mre11 and Rad50, heating at 60 °C for 15 min, and cooling to room temperature.

### Mre11 exonuclease assays

Exonuclease assays were performed with a dsDNA substrate that contained a 2-aminopurine (2-AP) at the eleventh nucleotide position from the 3′-end of one strand of the dsDNA (Exo11). The two DNA strands (strand 1: 5′-GGCGTGCCTTGGGCGCGC(2-AP)GCGGGCGGAG-3′ and strand 2: 5′-CTCCGCCCGCTGCGCGCCCAAGGCACGCC-3′; IDT) were annealed at equimolar concentrations. Each 30 µL exonuclease reaction was set up in a 1.5 mL microcentrifuge tube and contained 0.5 μM MR and 1 μM Exo11 dsDNA substrate in Reaction Buffer (50 mM HEPES, 150 mM NaCl, 0.1% PEG-6000, 2.5% glycerol, 1 mM TCEP, pH 7.0). Samples were made without co-factor, with 1 mM MnCl_2_, or with 1 mM MnCl_2_, 5 mM MgCl_2_, 1 mM ATP. After a 30 min incubation at 60 °C, the reactions were removed from the heat block, which stops the nuclease reaction, and 25 μL were transferred to a black 385-well plate. The 2-AP fluorescence resulting from Mre11 exonuclease activity was measured using a BioTek Synergy Neo2 multimode plate reader as previously described^34^.

### Mre11 endonuclease assay

A Cy3/BHQ2 fluorescence-based quantitative MR endonuclease assay was performed using a 17-nucleotide ssDNA substrate containing a Cy3 fluorophore at the 5′-end and a BHQ2 quencher at the 3′-end (5′-Cy3-TCTCTAGCAGTGGCGCC-BHQ2-3′; IDT). Note, Cy3 and BHQ2 are close enough on the intact ssDNA substrate that resonance energy transfer between Cy3 and BHQ2 results in low fluorescence signal. The experimental conditions and protocol were the same as described above for the 2-AP exonuclease assay except that 0.2 μM Cy3/BHQ-2 ssDNA was used as the substrate. Cy3 fluorescence resulting from endonuclease-cleaved substrate was measured as described previously^34^.

Qualitative endonuclease reactions on agarose gels were performed using 1 μg of ΦX174 single stranded virion circular DNA (New England Biolabs) as the substrate. 30 μL reactions containing 0.5 μM MR in Reaction Buffer were set up in 1.5 mL microcentrifuge tubes. Reactions were incubated at 60 °C without co-factor, with 1 mM MnCl_2_, or with 1 mM MnCl_2_, 5 mM MgCl_2_, 1 mM ATP. 6 µL time points were removed from the tube after 10, 20, and 30 min and quenched with 1% w/v SDS, 10 mM EDTA, and 0.5 mg/mL Proteinase K. Cleaved products were resolved on an 0.8% agarose gel, and visualized as previously described^34^.

### DNA binding experiments

ssDNA binding affinities were measured by monitoring the fluorescent polarization (FP) of a fluorescently labeled 40-mer DNA strand (5′-FAM-GTGTTCGGACTCTGCCTCAAGACGGTAGTCAACGTGCTTG-3′; IDT) as previously described^34^. For dsDNA binding assays, this fluorescently labeled DNA was annealed to an unlabeled complementary strand. *Pf* MR^NBD^ was titrated into 30 µL reactions containing 25 nM of either ssDNA or dsDNA substrate and 50 mM HEPES, 100 mM NaCl, 1 mM MnCl_2_, 0.1 mM EDTA, 1% glycerol, 1 mM TCEP, 0.1% PEG-6000, pH 7. After a 15 min incubation at room temperature, FAM FP was read in a Synergy Neo2 plate reader using the FP 485/530 filter. FP data were fit to the Hill equation for binding

$$FP= {FP}_{0}+\left({FP}_{max}- {FP}_{0}\right)\frac{{[DNA]}^{n}}{{[DNA]}^{n}+{K}_{D}^{n}}$$

where [DNA] is the concentration of DNA, K_D_ is the dissociation constant, n is the Hill coefficient, FP_0_ is the FP in the absence of protein, and FP_max_ is FP for maximum binding. Data points and error bars indicate the average and standard deviation of n = 3 experiments.

### NMR experiments

All NMR experiments on wild type and mutant samples were performed on a 600 MHz (14.1 T) Agilent DD2 NMR spectrometer equipped with a room temperature z-axis gradient HCN probe at 50 °C. Data sets were processed and analyzed with NMRPipe^59^ and CCPN analysis^60^. To determine the effects of mutation on DNA binding, samples were prepared with 1:4 molar excess of hairpin dsDNA (5′-CACGCACGTAGAAGCTTTTGCTTCTACGTGCGTGACT-3′; Sigma) or ssDNA (5′-TGTAGGTGCATTGCGTTTTTGCTTCTACGTGGTGAC-3′; Sigma). To analyze the effect of mutation or DNA binding, a weighted chemical shift perturbation (CSP) was calculated from the ^1^H (δ^H^) and ^13^C (δ^C^) chemical shifts according to

$$CSP= \sqrt{{\left(\frac{{\delta }_{i}^{H}-{\delta }_{j}^{H}}{{\sigma }_{H}}\right)}^{2}+{\left(\frac{{\delta }_{i}^{C}-{\delta }_{j}^{C}}{{\sigma }_{C}}\right)}^{2}}$$

where δ_i_ is the chemical shift value for wild type or DNA-free, δ_j_ is the chemical shift value for mutant or DNA-bound, and σ_H_ and σ_C_ are the standard deviations for methyl group specific δ^H^ and δ^C^ from the Biological Magnetic Resonance Data Bank.

Methyl group cross-correlated relaxation rates (η) were measured as described^43^. Pairs of triple (“forbidden”) and single (“allowed”) quantum data sets were collected in an interleaved manner for relaxation delay time (T) points of 2, 4, 6, 8, 10, 15, 20 and 25 ms. Ratio of peak intensities (Int_forbid_/Int_allow_) were fit to

$$\left|\frac{{Int}_{forbid}}{{Int}_{allow}}\right|=\frac{C (\eta \mathrm{tanh}(\sqrt{{\eta }^{2}+{\delta }^{2}} T))}{\sqrt{{\eta }^{2}+{\delta }^{2}}- \delta \mathrm{tanh}(\sqrt{{\eta }^{2}+{\delta }^{2}} T)}$$

where C is 0.75 and δ accounts for relaxation from external protons^43^. Errors in peak intensities were set to the noise of the spectra, and the reported errors in η are from the covariance of the fit.

### Methyl chemical shift covariance analysis

To perform the chemical shift covariance analysis (CHESCA)^38^, a weighted combined methyl chemical shift value (δ_methyl_) was calculated from the ^1^H (δ^H^) and ^13^C (δ^C^) chemical shifts as

$${\delta }_{methyl}= \frac{{\delta }^{H}}{{\sigma }_{H}}+ \frac{{\delta }^{C}}{{\sigma }_{C}}$$

where σ_H_ and σ_C_ are the standard deviations for methyl group specific δ^H^ and δ^C^ from the Biological Magnetic Resonance Data Bank. Side-chain methyl groups were selected for CHESCA using a median-absolute-deviation (MAD) approach^61^, which is used to find outliers from a distribution, with a cut-off of modified Z-score > 0.5. CHESCA was then performed on methyl groups with significant CSPs as a function of mutation with an in-house written python script (available upon request) that used the scientific python (scipy) library as previously described^42^. Briefly, unit-less inter-residue correlation distances^62,63^ were calculated between each methyl group using the set of δ_methyl_ values for the four Mre11^ND^ constructs. Correlation distances were then clustered using a complete linkage algorithm^64^. Finally, the four clusters were derived from a distance cut-off of 0.5 which was empirically determined based on the dendrogram in Fig. 3b.

### Yeast assays

C-terminal 13 × myc-tagged mre11 mutants were constructed in the integrated GAL10-HO cassette W303 *S. cerevisiae* strain (*MATa leu2::GAL-HO-LEU2 hml ∆hmr ∆RAD5*). The kanMX gene was cloned from pFA6a-kanMX vector (Addgene plasmid number 39296)^65^ and juxtaposed to the 3′ end of the *mre11* gene for geneticin (G418 di-sulfate salt from Sigma) antibiotic selectivity. The *mre11∆* strain was prepared by replacing the *mre11* open reading frame with the kanMX gene. The *sgs1*Δ strain was prepared by replacing the *sgs1* open reading frame with the *trp1* gene, which was cloned from the pRS414 vector (ATCC). Verification of the integrated genes was accomplished by PCR (*mre11* and *sgs1*) and western blot (mre11). The genotoxin sensitivity assay, NHEJ repair assay^48,49^, and real time PCR-based end resection assay^50,51^ were all performed as previously described^34^.

### Statistical analysis

Statistical analyses were performed using GraphPad Prism 8 (GraphPad Software Inc., San Diego, CA, USA). All data are presented as the mean and standard deviation (SD) of at least three replicates. As the exonuclease assay has only one variable, we used a one-way ANOVA with Dunnett correction. For all statistical tests, p < 0.01 was considered statistically significant.

## Supplementary Information

Supplementary Information.

## Data Availability

The datasets generated and/or analyzed during the current study are available from the corresponding author on reasonable request.
