# Supplementary material for: Biochemical and structural characterization of analogs of MRE11 breast cancer-associated mutant F237C
Source: Sci Rep. 2021 Mar 29;11:7089. doi: 10.1038/s41598-021-86552-0 (PMC8007570; doi:10.1038/s41598-021-86552-0)
Supplement: Supplementary file 1 — Supplementary Information. [file 41598_2021_86552_MOESM1_ESM.pdf]

Biochemical and structural characterization of analogs of MRE11 breast cancer-associated mutant F237C

Samiur Rahman, Mahtab Beikzadeh, Michael P. Latham*
Department of Chemistry and Biochemistry, Texas Tech University, Lubbock, TX, 79409-1061

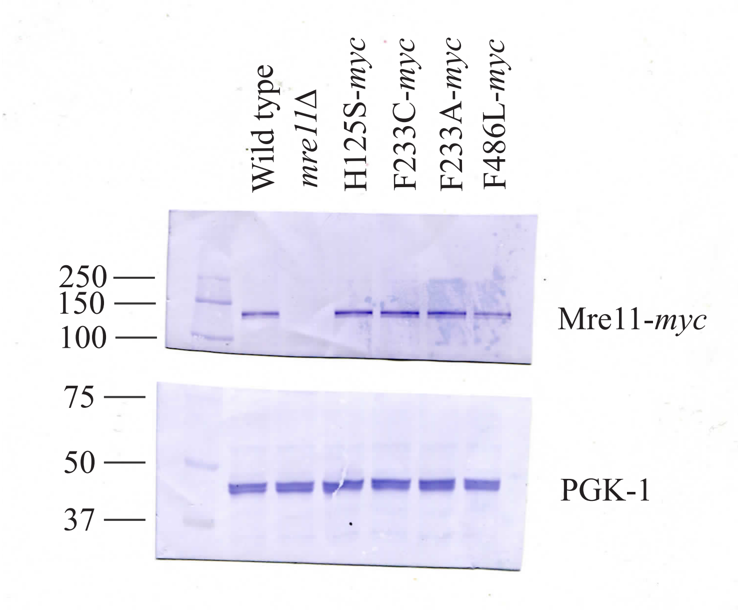

Supplementary Figure S1 | Complete gel for western blot shown in Figure 6A.
